# The Importance of Screening for Chagas Disease Against the Backdrop of Changing Epidemiology in the USA

**DOI:** 10.1007/s40475-022-00264-7

**Published:** 2022-09-10

**Authors:** Jennifer Ayres, Rachel Marcus, Claire J. Standley

**Affiliations:** 1 Department of Global Health, Georgetown University, Washington, DC USA; 2 Center for Global Health Science and Security, Georgetown University, Washington, DC USA; 3 Latin American Society of Chagas, Washington, DC USA; 4 Medstar Union Memorial Hospital, Baltimore, MD USA; 5 Heidelberg Institute of Global Health, University of Heidelberg, Heidelberg, Germany

**Keywords:** Chagas disease, *Trypanosoma cruzi*, Neglected tropical diseases, Zoonotic diseases, Infectious disease screening and detection, United States of America

## Abstract

**Purpose of Review:**

This review seeks to identify factors contributing to the changing epidemiology of Chagas disease in the United States of America (US). By showcasing screening programs for Chagas disease that currently exist in endemic and non-endemic settings, we make recommendations for expanding access to Chagas disease diagnosis and care in the US.

**Recent Findings:**

Several factors including but not limited to increasing migration, climate change, rapid population growth, growing urbanization, changing transportation patterns, and rising poverty are thought to contribute to changes in the epidemiology of Chagas disease in the US. Outlined are some examples of successful screening programs for Chagas disease in other countries as well as in some areas of the US, notably those which focus on screening high-risk populations and are linked to affordable and effective treatment options.

**Summary:**

Given concerns that Chagas disease prevalence and even risk of transmission may be increasing in the US, there is a need for improving detection and treatment of the disease. There are many successful screening programs in place that can be replicated and/or expanded upon in the US. Specifically, we propose integrating Chagas disease into relevant clinical guidelines, particularly in cardiology and obstetrics/gynecology, and using advocacy as a tool to raise awareness of Chagas disease.

## Introduction

Chagas disease is a neglected tropical disease that affects approximately 6–7 million people globally [1]. Caused by the parasite *Trypanosoma cruzi*, infection is predominantly spread through contact with *T. cruzi*-infected triatomine bugs, sometimes referred to as “kissing bugs.” These bugs are endemic throughout most tropical and sub-tropical regions of the Americas, although they also range into some temperate areas. While historically the majority of infections occurred in rural areas of Mexico, Central America, and South America, there are increasing accounts of peri-urban and urban transmission [2]. Infection can also occur transplacentally, through contaminated blood transfusions and organ transplants, as well as via consumption of food or drink contaminated with *T. cruzi *[3, 4].

Although the United States (US) is not generally considered to be Chagas disease endemic, vector-borne transmission of the disease occurs sporadically, and reviews of published case reports suggest that perhaps autochthonous transmission may be more common than previously thought [5–7]. Triatomine bugs have been detected in more than half of the continuous lower 48 states of the US, with larger populations and higher species diversity in the southernmost states [8]. Enzootic *T. cruzi* transmission occurs frequently in the southern US, with recent studies observing high incidence rates of new infections among kennel-housed domestic dogs [9•]. Other domestic, peri-domestic, and wild animal species, including rats, raccoons, opossums, and coyotes, have also been identified as infected with *T. cruzi* in the US [10].

Currently, the epidemiology of Chagas disease in the US may be changing due to factors such as continuous immigration and expanded familial ties between the US and endemic regions of Latin America; an increase in the number of people moving to southern regions in the US, meaning more encroachment on triatomine habitat and increased human-triatomine contact; and climate change, which could expand the habitat range of triatomine vectors. There is a need to improve screening for and detection of new as well as existing cases of Chagas disease, in order to address the health needs of affected populations, to monitor these potential changes in Chagas epidemiology, and establish suitable risk mitigation measures. In this review, we aim to (i) describe current and predicted patterns of Chagas disease epidemiology and risk in the US; (ii) present examples of how screening and treatment programs have been established in other endemic and non-endemic countries; and (iii) review current initiatives, challenges, and opportunities for expanding access to Chagas disease diagnosis and care across different US settings.

## Dynamics of Epidemiology and Distribution of Chagas Disease in the US

Estimates suggest that the majority of *T. cruzi*-infected individuals in the US are immigrants from other Chagas-endemic regions of Latin America [11]. As Latin American migration to the US has increased over the past two decades, it is suggested that so have the number of imported Chagas disease cases, especially in southern states [12, 13, 14•]. An assessment on the prevalence of Chagas disease based on combining demographic information from the American Community Survey with Chagas disease prevalence estimates from different Latin American countries suggested that currently there are at least 300,000 people with the disease in the US [15••]. It is important to note that these data likely underestimate the true number of individuals in the US living with Chagas disease, since the number of undocumented immigrants from endemic countries is unknown, and only seven states list Chagas disease as a reportable or notifiable condition to the US Centers for Disease Control and Prevention (CDC) [16]. Analysis of *T. cruzi* seropositivity in donated blood from 2007 to 2015 revealed no national-level changes in rates, but did suggest presence of five likely autochthonous infections [17]. However, estimates from screening of donated blood are likely to underestimate Chagas disease prevalence, especially in migrant groups [18].

Another factor that may be impacting Chagas disease epidemiology and distribution in the US, and will likely have a more profound future effect, is climate change [19•]. Climate change has been shown to have an effect on Chagas disease exposure risk across a number of variables, including via changes to precipitation, temperature, and relative humidity. These factors influence the life cycles of triatomine vector species, leading to changes in range, distribution, and behavior that can affect the risk of Chagas disease transmission [20]. A systematic review of projected climate change impacts on Chagas disease in the Americas concluded that distributions of most epidemiologically important triatomine species in Central and South America will decrease in geographical extent, potentially lowering the risk of Chagas disease in these areas [19•]. Conversely, range expansions are anticipated to occur in Mexico and parts of the US, most notably with new suitable habitats for triatomine persistence extending northwards in the US [21]. To this end, changing climatic patterns may reduce the risk of imported cases of Chagas disease, while increasing the threat from autochthonous infection.

In addition to human migration and climate change, other factors related to modernization and globalization are known to contribute to the risk of emergence and re-emergence of neglected diseases such as Chagas disease in the US. This includes rapid population growth; growing urbanization; changing transportation patterns; and the overall rise in poverty in numerous areas throughout the country [20]. These factors may also influence the availability, distribution, and population sizes of non-human Chagas disease hosts, including opossums, raccoons, coyotes, and other mammalian species that are particularly or increasingly well-adapted to human-mediated environments [12]. Dogs, and potentially all canid species, moreover can experience morbidity from infection with *T. cruzi*, thus demonstrating the potential veterinary impact of changing Chagas disease epidemiology within the US [22]. The interplay between all of these factors may contribute to increased prevalence of Chagas disease in the US, especially among specific populations.

## Surveillance Efforts for Chagas Disease in Other Countries

### Overview of Screening Programs Abroad

Chagas disease screening programs are an effective way to assess prevalence as well as diagnose and administer treatment to those who need it. Increasing numbers of studies, especially in non-endemic countries, have identified Chagas disease as a potential public health concern among migrants from Latin America, but the importance of Chagas disease as a health issue for intra-regional migration within Latin America has also been recognized [18, 23–25]. To this end, there are numerous screening programs that exist for Chagas disease abroad including but not limited to a neonatal screening program in Guatemala [26]; screening for pregnant people from Latin America in Spain [27•]; screening at the primary healthcare level for Bolivian immigrants in Brazil [28]; efforts to combat Chagas disease in the UK [29, 30]; Argentina’s national program to detect and treat *T. cruzi* infected infants [31]; and improving access to testing for Chagas disease in Colombia [32].

Here, we describe several of these screening programs in further depth, as elements of these programs are relevant to the US context. It is important to note that the US and many other countries, but especially endemic settings in Latin America, face some similar challenges in addressing Chagas disease. These include increasing awareness of Chagas disease without increasing the stigma surrounding it; improving data collection to better understand the burden of disease; preventing transplacental transmission of the disease; and stopping the development of severe cardiomyopathy [33].

### Neonatal Chagas Disease Screening Program in Guatemala

In a rural community in Guatemala, congenital screening was done at the primary healthcare level to diagnose newborns and infants with Chagas disease. First, 228 pregnant women attending a local health clinic were screened for *T. cruzi* infection via a rapid test. If a woman was found to be seropositive, her newborn was given either a parasitic test (the preferred method) or an antibody test if taken ten or more months after birth to allow clearance of maternal antibodies from the neonate’s blood. The parasitological test consisted of microscopic examination of the infant’s blood and required only a lancet, slide, and microscope. Because the parasitic load in an infected infant rises in the first month after birth, the sensitivity of this test is between forty and 60%; however, the specificity is believed to be high enough that an infant testing positive is treated for Chagas disease. Treatment for infected mothers and their infants was given immediately after diagnosis [26]. Eight of the 228 women (3.9%) were diagnosed with Chagas disease as a result of the program, and seven of eight newborns born to seropositive mothers were then parasitologically screened for *T. cruzi* infection; no newborns were found to be positive for *T. cruzi* [26]. This screening program was both simple and low cost, and demonstrated that community-based neonatal screening can be successfully implemented in a rural setting. The program was supplemented with training for midwives integrated into existing midwife training sessions, and this was important as it increased awareness of the disease [26]. Given that Chagas disease remains endemic in Guatemala, the program serves as an important example of effective community level screening that could be replicated in the US.

### Prenatal Chagas Disease Screening in Spain

Spain has the second largest population of Latin American immigrants in the world, behind only the US [27•]. In 2005, the country implemented a mandatory screening of blood donors at risk for infection with *T. cruzi*. The mandate targeted donors born in endemic areas, donors whose mothers were born in endemic areas, and individuals who received blood transfusions in endemic areas [27•]. Chagas disease screening for all pregnant women from Latin America has been mandated in Valencia and Catalonia, two autonomous communities in Spain. Through the screening programs, pregnant women and their newborns are able to get screened for *T. cruzi* infection and, if needed, receive treatment. If the mother is found to be infected with the parasite, screening and treatment are extended to her other children who might unknowingly also have the disease [34]. Data are limited on the success of the mandatory screening programs of pregnant women in Valencia and Catalonia, but it is known that during 2009 and 2010, 226 pregnant women in Valencia were diagnosed with Chagas disease, and in 2011, 179 pregnant women in Catalonia were diagnosed with the disease [35, 36]. Despite the successful detection of hundreds of Chagas disease cases in this mandatory screening program, systemic detection of transplacental Chagas disease is not performed in the rest of the country [27•]. In a study of congenital *T. cruzi* transmission in 122 seropositive pregnant women from endemic areas now living in Madrid, 2.75% of infants were found to be infected with the parasite, illustrating the need to screening for Chagas disease among pregnant women from endemic areas [37].

Preventive measures at the national level such as wide scale screening have yet to be implemented in Spain or elsewhere in Europe. As in most non-endemic regions, health professionals in Europe generally lack experience with detecting and managing Chagas disease among their patients, so protocols addressing this disease have been slow to develop. In addition, ensuring that the people and communities at risk have sufficient access to screening programs is difficult because few institutions offer this type of screening, most of which are located in major urban areas [38, 39].

### Efforts to Combat Chagas Disease in the UK

Latin Americans are the second fastest growing migrant population in London [29], and it is imperative that Chagas disease diagnosis, prevention, and disease management be addressed by the UK healthcare system [29]. To address this emerging challenge, in 1999, the UK implemented systematic screening of at-risk blood and organ donors to prevent the transmission of *T. cruzi* infection [29]. In 2010, Public Health England (PHE) developed the Migrant Health Guide, which is a resource for healthcare professionals working with migrant populations. The guide is meant to raise awareness of Chagas disease and other issues specifically affecting migrant populations. Furthermore, the guide recommends that high-risk women who are pregnant or are of child-bearing age be offered serological testing for *T. cruzi* infection [29]. The majority of Latin Americans migrants living in the UK reside in London, and between the years of 2001 and 2014, 41 cases of *T. cruzi* infections were detected in London [40, 41]. Nevertheless, physicians and other medical health professionals continue to lack awareness of Chagas disease, and routine screening of at-risk women in the UK is not taking place to the extent that it should be [27•].

The Chagas Hub is another initiative in the UK in order to strengthen screening and treatment services for Chagas disease in the country [30]. The initiative is a collaboration between healthcare professionals, researchers, advocates, and members of the Latin American community, and its goals include improving clinical services such as creating a dedicated Chagas clinic in the UK; conducting research into the epidemiology and clinical manifestations of Chagas disease in London; and raising awareness of Chagas disease in Latin American communities through public engagement activities [30].

## Chagas Disease Screening Efforts in the US

### Overview of Screening Programs in the US

No national screening program exists for Chagas disease in the US, nor is there active surveillance for the disease does. *T. cruzi* infections are most often detected through blood donor screening, which was implemented at the national level in 2007 [42]. Blood donor screening is limited in its reach, and has diagnosed just 2435 of the estimated 300,000 + infections within the country, as of 26 October 2019 [10, 16]. In addition, Chagas disease is only notifiable in seven US states—Arizona, Arkansas, Louisiana, Mississippi, Tennessee, Texas, and Utah—with the state of Texas alone implementing active surveillance of the disease [42]. Recently, Chagas disease was also added as a reportable disease in Los Angeles County [43].

There have been several efforts to screen for Chagas disease in the US, including but not limited to a community-based screening study in the Washington metropolitan area; a Chagas disease screening program at the East Boston Neighborhood Health Center; and a community-based screening program at the Center for Excellence for Chagas Disease in Los Angeles.

### Community-Based Screening Study in Washington, DC

From February 2016 to July of 2018, Castro and colleagues [44] collected data on the seroprevalence of Chagas disease in Washington, DC. This was done through a community-based screening program of Latin American immigrants over the age of 18 years living in Washington, DC, who were born in a country where Chagas disease is endemic. The authors found that a *T. cruzi* seroprevalence of 3.8% (*N* = 1514). Results from this study found the highest *T. cruzi* presence in persons from Bolivia, which has the highest national prevalence of Chagas disease in the world, and that a history of having seen the vector in endemic areas was a risk factor for infection [44].

### Chagas Disease Screening Program in East Boston

At the East Boston Neighborhood Health Center (EBNHC) from March 2017 to May 2020, Chagas disease screening was integrated into the primary healthcare setting through the Strong Hearts pilot project [45]. Specifically, this project conducted screening for Chagas disease among patients who were 50 years old or older and had lived in Mexico, Central America, or South America for at least 6 months during their lifetime. In total, the program tested 8142 patients for *T. cruzi* infection, and found a prevalence of nearly 1% (76 positive individuals) [46].

### Community-Based Screening at the Center for Excellence for Chagas Disease in Los Angeles

The Center of Excellence for Chagas Disease (CECD) in Los Angeles is currently the only center in the US that focuses on the diagnosis and treatment of Chagas disease [47]. Between April 2008 and May 2014, the CECD screened 4755 residents in LA county who were born in Latin America. Among these individuals, 59 (1.24%) were found to have Chagas disease. This was the largest Chagas disease screening ever carried out in the US. Extrapolations from this study suggest that more than 30,000 people in LA county could be infected with *T. cruzi*, highlighting the importance of addressing this issue and screening for Chagas disease in the US [48].

A follow-up study was conducted at the CECD to assess the prevalence of Chagas disease among family members of those who had previously been diagnosed with the disease. In this study, 189 relatives of 86 existing patients identified through either the community-based screening program or referral were screened for Chagas disease. Participants varied in age from newborns to adults, and nearly half of the respondents were born in the US. Although the majority of these respondents (73.0%) had a parent who was diagnosed with Chagas disease, others were siblings, spouses, or parents themselves. The overall prevalence rate of Chagas disease among the close relatives who were screened in this study was 7.4%, suggesting that having a family member previously diagnosed with the disease increases a person’s statistical likelihood of being infected already [49].

Both studies which took place at the CECD found country of origin to be a risk factor for *T. cruzi* infection. In the community-based screening program, individuals from El Salvador had the highest *T. cruzi* infection rate (3.45%), and were 6.2 times more likely to have Chagas disease compared to other Latin Americans from countries such as Mexico and Guatemala [48]. Similarly, family members who were Salvadoran also had the highest overall prevalence rate (16.4%) among those who participated in the second study [49].

Results from the original study also confirmed that there is a lack of Chagas disease awareness among those infected. Using data from 2677 participants who completed a questionnaire on Chagas disease knowledge and awareness, it was found that despite many being aware of the triatomine bug vectors, they were not as familiar with the disease itself. Specifically, 86% of the participants had not heard of Chagas disease before, and of those who were familiar with the disease, 81% thought it was not a serious disease. These findings clearly illustrate the need to educate those at risk of exposure about the disease, including the health risks of Chagas disease if left untreated [50].

## Recommendations for Improving Screening for Chagas Disease in the US

Our review of the existing literature on Chagas disease epidemiology in the US combined with our research into successful screening and control programs domestically and abroad leads us to a number of recommendations for improving detection and treatment of Chagas disease in the US.

### Integrate Chagas Disease into Relevant Clinical Guidelines

Chagas disease awareness campaigns raising awareness among patients as well as the medical and public health communities could have profound impact [51••]. Chagas disease remains neglected as a differential diagnostic, for example in cardiac patients, even in the presence of other risk factors, limiting opportunities for case detection and treatment [52]. While efforts have been made to raise awareness among patients and provide additional resources to clinicians [53], information regarding risk factors, testing procedures, and case management steps must be integrated into relevant clinical guidelines. Such guidelines exist in endemic countries and at a regional level but have not yet been adapted and adopted by American medical professional associations [54–56]. One barrier to uptake of such guidelines has historically been the lack of data on Chagas disease prevalence, as well as the overall rarity of the disease at a population level. We argue that the growing literature describing the occurrence of Chagas disease among numerous populations in the US, as well as the characterization of groups at elevated risk, overcomes this burden of proof and squarely demonstrates the value in urgently updating relevant medical guidelines to account for Chagas disease.

Cardiology and obstetrics/gynecology are two specialties where updated guidelines would be particularly beneficial. Chagas disease is a substantial contributor to heart disease in Latin American immigrant populations living in the US, and is also associated with poorer health outcomes in those patients [57••, 58]. Unfortunately, direct treatment success in patients with advanced cardiac complications from Chagas disease is limited, and the lack of Chagas-specific randomized trials further reduces the availability of “gold standard” data upon which to generate guidelines for patient care. However, sufficient evidence does exist to suggest that existing guidelines could at least be updated and clarified, leading to earlier and more accurate detection, and potentially providing opportunities for further research into case management options.

### Antenatal Screening

The recommendation of antenatal screening is echoed repeatedly in most global health publications relating to Chagas disease, and rightly so, treatment of congenital Chagas disease within the first year of life has an over 90% success rate. The effort-benefit tradeoff is simple and implementation is highly feasible. As such, screening of pregnant women is a key opportunity to save lives, in both endemic and non-endemic settings, as shown in examples from Guatemala and Spain. CDC already provides algorithms for the evaluation of Chagas disease in pregnant women as well as in infants [2].

### Operational Research to Link Delivery of Interrelated Services

The availability of interrelated interventions such as screening and treatment suggests the need for operational research to better understand how to reach at-risk populations, and the most efficient and effective methods of service delivery. Despite being more prevalent than previously assumed, and with likely expanding risk due to climate and demographic change, Chagas disease diagnosis remains rare in the US. To this end, to be cost-effective, interventions will need to be strategic, targeted, and leverage existing healthcare infrastructure. While studies in Spain have demonstrated the cost-effectiveness of screening pregnant women [27•], such calculations are more complicated in the US, with its fragmented and heavily privatized healthcare system. Thus, although some analyses show substantial potential cost–benefit to maternal screening, for example, these estimates need to be considered alongside other aspects of operational research [59].

### Advocacy

Advocacy, such as via the Hispanic Caucus in Congress and allied state-level bodies, may also prove to be a powerful tool in raising awareness and promoting uptake of available interventions, particularly if fears around costs of seeking care or documentation status can be assuaged [14•, 60, 61]. This type of political awareness-raising might also be beneficial for encouraging more states to legislate Chagas disease as reportable or notifiable to CDC, increasing the availability of data on incidence and prevalence, and further facilitating advocacy.

### Increased Access to Diagnostic Testing

A key barrier often cited with respect to increasing advocacy, as well scaling up screening and treatment programs, relates to the difficulties of diagnosing *T. cruzi* infection. Domestically, CDC’s Infectious Disease Laboratory provides free of cost confirmatory diagnosis if an initial screening test has already been performed [62]. The reliance on a single national laboratory has the potential to create a diagnostic bottleneck, as seen during the COVID-19 pandemic when the CDC stopped offering testing for many diseases, including both serological and molecular diagnosis of Chagas disease [63]. With the one laboratory offering testing gone, there was no way to diagnose the disease in the US for over a year, removing a critical resource for patient diagnosis, especially for not-for-profit community screening and treatment initiatives focused on under- or uninsured populations.

It is important to note that CDC’s involvement in diagnostics was historically tied closely to patient case management. Prior to 2018, a positive confirmation of Chagas disease diagnosis from CDC was necessary to be able to obtain medication to treat the disease. CDC worked closely with care providers to manage treatment in affected patients. In 2017, the Food and Drug Administration approved the use of benznidazole for treatment of Chagas disease, and allowed commercial access in 2018. While this appears to have increased the number of individuals in the US who received treatment for Chagas disease, the lack of institutional oversight has raised concerns over improper or contraindicated use of benznidazole [64]. On the other hand, the decoupling of treatment from CDC laboratory confirmation provides a useful opportunity for expansion of laboratory testing capacity, for example, via academic institutions that could serve both as reference centers for diagnostics as well as private sector companies contributing to research and development of new diagnostic tools [65, 66].

## Conclusions

Based on the current epidemiology of Chagas disease in the US, as well as various factors likely to contribute to an increased risk of disease domestically, the time has come to take Chagas disease seriously in the US. There is substantial evidence documenting its significance as a public health challenge, with disproportionate impact in migrant communities, such that concerted national and state-level efforts are warranted to increase advocacy, awareness, as well as affordable and accessible opportunities for diagnosis and treatment. Screening programs, particularly targeting women of reproductive age as well as cardiac patients among higher-risk groups, could prove to be particularly effective at identifying new cases and providing appropriate interventions, as have been demonstrated in several other countries around the world. We suggest that being considered as an endemic country for Chagas disease might provide an important catalyst for encouraging US leadership in future efforts to develop, implement, and evaluate approaches for expanding access to screening as well as diagnostics, treatment, and social support for affected communities.
